# MERS–Related Betacoronavirus in *Vespertilio superans* Bats, China

**DOI:** 10.3201/eid2007.140318

**Published:** 2014-07

**Authors:** Li Yang, Zhiqiang Wu, Xianwen Ren, Fan Yang, Junpeng Zhang, Guimei He, Jie Dong, Lilian Sun, Yafang Zhu, Shuyi Zhang, Qi Jin

**Affiliations:** Ministry of Health Key Laboratory of Systems Biology of Pathogens, Beijing, China (L. Yang, Z. Wu, X. Ren, F. Yang, J. Dong, L. Sun, Y. Zhu, Q. Jin);; Institute of Pathogen Biology, Beijing (L. Yang, Z. Wu, X. Ren, F. Yang, J. Dong, L. Sun, Y. Zhu, Q. Jin);; East China Normal University, Shanghai, China (J. Zhang, G. He, S. Zhang)

**Keywords:** coronavirus, Middle East respiratory syndrome coronavirus, MERS-CoV, Vespertilio superans, bat, reservoir, sequencing, lineage, betacoronaviruses, viruses, MERS–related betacoronavirus, Middle East respiratory syndrome coronavirus–related betacoronavirus, China, lineage C betacoronavirus

**To the Editor:** Middle East respiratory syndrome coronavirus (MERS-CoV), a novel lineage C betacoronavirus, was first described in September 2012, and by April 16, 2014, the virus had caused 238 infections and 92 deaths in humans worldwide (*1*). Antibodies against MERS-CoV in dromedary camels were recently reported (*2*), as was the full genome of MERS-CoV from dromedary camels (*3*). Finding the natural reservoir of MERS-CoV is fundamental to our ability to control transmission of this virus to humans (*4*).

We report a novel lineage C betacoronavirus identified from *Vespertilio superans* bats in China. The full-length genome of this betacoronavirus showed close genetic relationship with MERS-CoV. Together with other evidence of MERS-CoV–related viruses in bats (*5*–*8*), our findings suggest that bats might be the natural reservoirs of MERS-related CoVs.

In June 2013, we collected anal swab samples from 32 *V. superans* bats from southwestern China. A small proportion of each sample was pooled (without barcoding) and processed by using virus particle–protected nucleic acid purification and sequence-independent PCR for next-generation sequencing analysis with the Illumina (Solexa) Genome Analyzer II (Illumina, San Diego, CA, USA). Redundant reads were filtered, as described (*9*), from the raw sequencing reads generated by the genome analyzer and then aligned with the nonredundant protein database of the National Center for Biotechnology Information (ftp://ftp.ncbi.nlm.nih.gov/blast/db/) by using BLAST (http://blast.ncbi.nlm.nih.gov). The taxonomy of these aligned reads was parsed by using MEGAN 4 (http://ab.inf.uni-tuebingen.de/software/megan/).

On the basis of the BLAST results, 8,751,354 sequence reads 81 nt in length were aligned with the protein sequences of the nonredundant protein database: 72,084 of the reads were uniquely matched with virus proteins. Of these 72,084 reads, 32,365 were assigned to the family *Coronaviridae*, primarily to lineage C of the genus *Betacoronavirus*, and found to share 60%–97% aa identity with MERS-CoV.

The MERS-CoV–related reads were extracted and assembled by using SeqMan software from the Lasergene 7.1.0 program (DNASTAR, Madison, WI, USA), resulting in a draft CoV genome. Reverse transcription PCR selective for the partial RNA-dependent RNA polymerase (RdRp) gene of this novel lineage C betacoronavirus suggested that 5 of the 32 samples (≈16%) were positive for the novel betacoronavirus, and the PCR amplicons shared >98% nt identity with each other. Using a set of overlapped nested PCRs and the rapid amplification of cDNA ends method, we determined the full-length genome of 1 strain of this *V. superans* bat–derived betacoronavirus (referred to as BtVs-BetaCoV/SC2013, GenBank accession no. KJ473821).

The betacoronavirus strain had a genome length of 30,413 nt, excluding the 3′ poly (A) tails, and a G+C content of 43.1%. Pairwise genome sequence alignment, conducted by the EMBOSS Needle software (http://www.ebi.ac.uk/Tools/psa/emboss_needle/) with default parameters, suggested that the genome sequence of BtVs-BetaCoV/SC2013 showed 75.7% nt identity with that of human MERS-CoV (hCoV-MERS); this shared identity is higher than that for other lineage C betacoronaviruses (from bats and hedgehogs) with full genomes available. hCoV-MERS showed 69.9% nt identity with bat CoV (BtCoV) HKU4-1, 70.1% nt identity with BtCoV-HKU5-1, and 69.6% nt identity with hedgehog CoV EriCoV-2012–174.

Compared with those lineage C betacoronaviruses, which had an 816-bp partial RdRp sequence fragment available, BtVs-BetaCoV/SC2013 shared 96.7 % aa identity with hCoV-MERS. *Pipistrellus* BtCoVs found in Europe (BtCoV-8-724, BtCoV-8-691, BtCoV-UKR-G17) shared 98.2 % aa identity with hCoV-MERS, and *Eptesicus* BtCoV found in Italy (BtCoV-ITA26/384/2012) and other lineage C betacoronaviruses shared 96.3 % aa and <95% aa identity, respectively, with hCoV-MERS.

To clarify the evolutionary relationship between BtVs-BetaCoV/SC2013 and other lineage C betacoronaviruses, we performed phylogenetic analyses based on the deduced RdRp and the spike, envelope, membrane, and nucleocapsid proteins by using MEGA5 (http://www.megasoftware.net/) (Figure; Technical Appendix). For RdRp and the envelope, membrane, and nucleocapsid proteins, BtVs-BetaCoV/SC2013 always clustered with hCoV-MERS with short branch lengths, reflecting their high sequence similarities.

**Figure:**
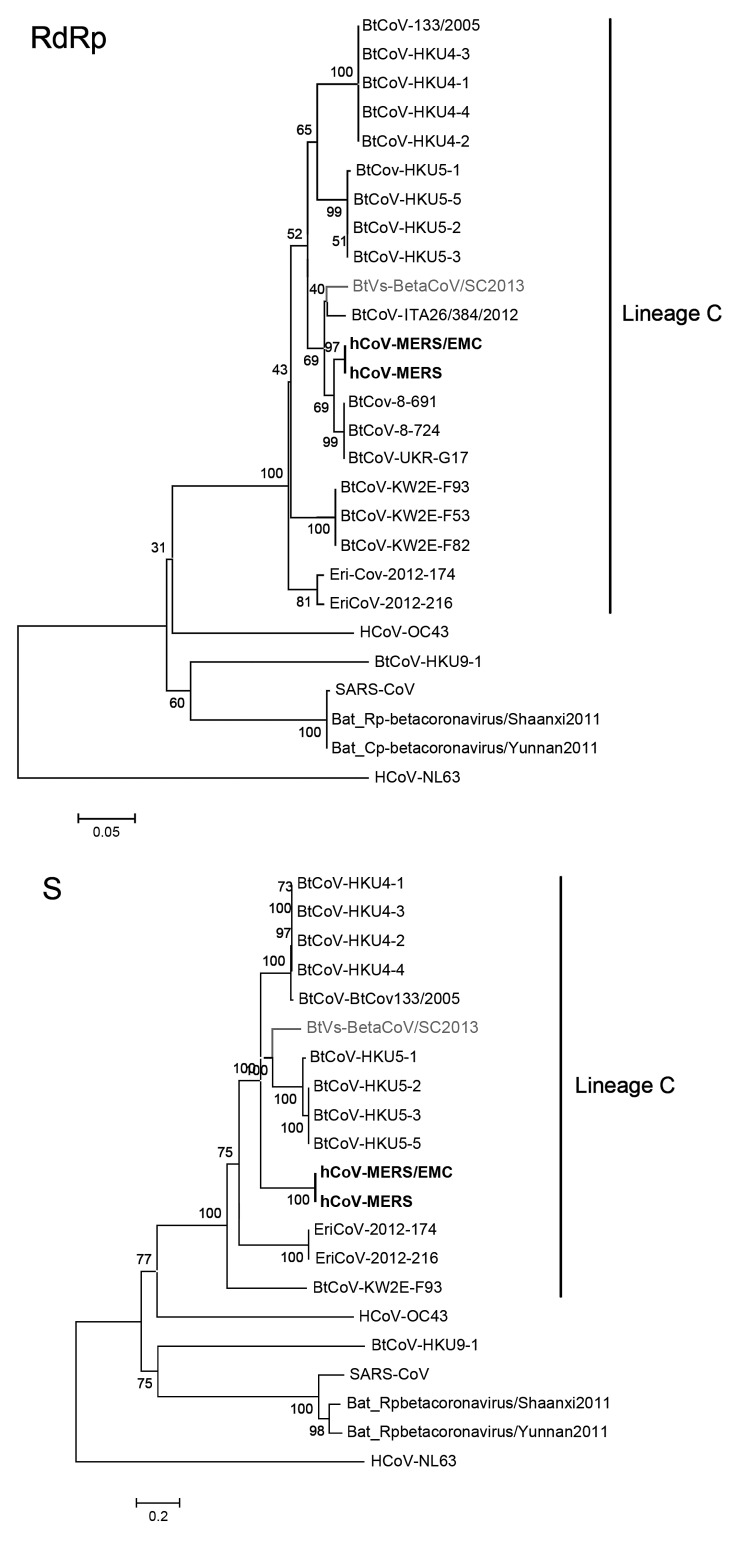
Phylogenetic trees based on the deduced amino acid sequences of the partial RNA-dependent RNA polymerase (RdRp; an 816-nt sequence fragment corresponding to positions 14817–15632 in human Middle East respiratory syndrome coronavirus [hCoV-MERS; KF192507]) and complete spike (S) protein. The novel virus is shown in gray, and hCoV-MERS is shown in bold. The following coronaviruses were used (GenBank accession numbers are shown in parentheses): severe acute respiratory syndrome coronavirus (SARS-CoV; NC004718), Bat Rp-coronavirus/Shaanxi2011(JX993987), Bat Cp-coronavirus/Yunnan2011(JX993988), Bat coronavirus HKU9-1 (BtCoV-HKU9-1; EF065513), BtCoV-133/2005(NC008315), BtCoV-HKU4-1 (EF065505), BtCoV-HKU4-2 (EF065506), BtCoV-HKU4-3 (EF065507), BtCoV-HKU4-4 (EF065508), BtCoV-HKU5-1 (EF065509), BtCoV-HKU5-2 (EF065510), BtCoV-HKU5-3 (EF065511), BtCoV-HKU5-5 (EF065512), BtCoV-ITA26/384/2012 (KF312399), BtCoV-KW2E-F82 (JX899382), BtCoV-KW2E-F93 (JX899383), BtCoV-KW2E-F53 (JX899384), BtCoV-8–724 (KC243390), BtCoV-8–691 (KC243391), BtCoV-UKR-G17 (KC243392), Human betacoronavirus 2c EMC/2012 (hCoV-MERS/EMC; JX869059), hCoV-OC43 (NC005147), hCoV-NL63 (NC005831), Betacoronavirus ErinaceusCoV/2012-174 (EriCoV-2012-174; KC545383), and EriCoV-2012-216 (KC545386). Scale bar indicates genetic distance estimated by using WAG+G model for the RdRp and WAG+G+F model for the S protein implemented in MEGA5 (http://www.megasoftware.net/).

In the spike protein phylogenetic tree, BtVs-BetaCoV/SC2013 clustered with a clade defined by BtCoV-HKU5, with which it shares 74.8% aa identity. The spike proteins of hCoV-MERS form a sister clade of the clade defined by HKU5 BtCoVs and BtVs-betaCoV/SC2013, and the spike proteins share 69.0% aa identity with BtVs-betaCoV/SC2013. Spike proteins of BtVs-BetaCoV/SC2013, HKU5 BtCoVs, HKU4 BtCoVs, and hCoV-MERS, rather than EriCoV-2012-174, EriCoV-2012-216, and BtCoV-KW2E-F93, form a super clade. Spike protein is the critical factor for receptor recognition, binding, and cellular entry of CoVs in different host species (*10*), which may explain why the spike proteins in our study were relatively conserved within the same host species.

We identified a novel lineage C betacoronavirus from a *V. superans* bat and determined its full-length genome sequence. This novel betacoronavirus represents one of the most MERS-like CoVs that have been identified in bats as of the end of March 2014. The full-length genome sequence of the novel virus showed a closer genetic relationship with hCoV-MERS and camel MERS-CoV than with any other fully sequenced lineage C betacoronaviruses previously identified in bats or hedgehogs. Further studies of CoVs from more bat species worldwide may, therefore, help provide additional clues to the origins of pathogenic hCoV-MERS.

Technical Appendix Phylogenetic trees of a novel Middle East respiratory syndrome–related coronavirus, human Middle East respiratory syndrome coronaviruses, severe acute respiratory syndrome virus, and various other coronaviruses.
