# Supplementary material for: MERS–Related Betacoronavirus in Vespertilio superans Bats, China
Source: Emerg Infect Dis. 2014 Jul;20(7):1260–2. doi: 10.3201/eid2007.140318 (PMC4073873; doi:10.3201/eid2007.140318)
Supplement: Technical Appendix — Phylogenetic trees of a novel Middle East respiratory syndrome–related coronavirus, human Middle East respiratory syndrome coronaviruses, severe acute respiratory syndrome virus, and various other coronaviruses. [file 14-0318-Techapp-s1.pdf]

Article DOI: http://dx.doi.org/10.3201/eid2007.140318

MERS–Related Betacoronavirus in *Vespertilio superans* Bats, China

Technical Appendix

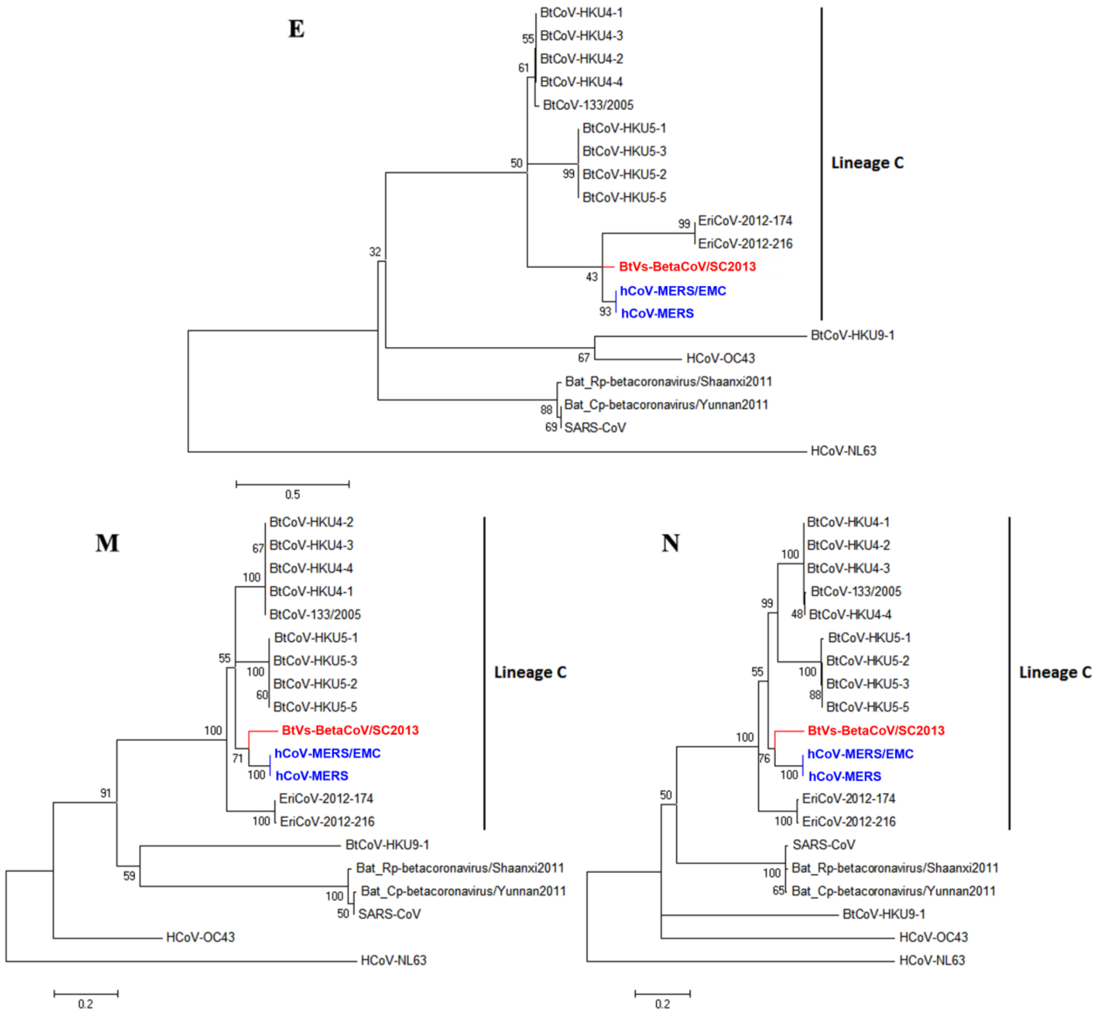

Technical Appendix Figure. Phylogenetic trees based on the deduced amino acid sequences of the complete envelope (E), membrane (M), and nucleocapsid (N) proteins. The novel virus is shown in red, and human Middle East respiratory syndrome coronaviruses (MERS-CoVs) in blue. SARS, severe acute respiratory syndrome; BtCoV, bat CoV; HCoV, human CoV; EriCoV, Erinaceus CoV. Scale bar indicates genetic distance estimated by using the WAG+G model implemented in MEGA5 (www.megasoftware.net).
